# Accuracy of gadolinium-enhanced cardiovascular magnetic resonance in the diagnosis of cardiac sarcoidosis

**DOI:** 10.1186/1532-429X-11-S1-P285

**Published:** 2009-01-28

**Authors:** Leonidas Tzogias, Mario Njeim, Gurjit Singh, Claudio Schuger, Khalid Nour, Milan V Pantelic, Mouaz H Al-Mallah

**Affiliations:** Henry Ford Hospital, Detroit, MI USA

**Keywords:** Cardiovascular Magnetic Resonance, Sarcoidosis, Late Gadolinium Enhancement, Diagnostic Yield, Cardiac Involvement

## Introduction

Cardiac arrhythmias are highly prevalent in cardiac sarcoidosis, however identification of cardiac involvement in sarcoidosis remains challenging. The role of gadolinium-enhanced cardiovascular magnetic resonance (CMR) in the diagnosis of Cardiac sarcoidosis (CS) is not well established.

## Purpose

This study analyzed the accuracy of CMR in the diagnosis of CS among patients with findings suggestive of CS.

## Methods

We performed gadolinium-enhanced CMR in 50 consecutive sarcoidosis patients (63% females) who presented with symptoms suggestive of cardiac involvement. Imaging protocol included steady-state acquisition cine imaging, T2-weighted black blood imaging and post-contrast delayed enhancement on a 1.5-T scanner. The diagnostic accuracy of CMR for CS was determined using the Japanese criteria (JMH) as the gold standard.

## Results

The diagnosis of CS was made according to JMH criteria in 9 of 50 patients (18%). CMR revealed patchy late gadolinium enhancement (LGE), mostly involving basal and lateral segments in 12 patients. The presenting symptoms of these patients were AV block, ventricular arrhythmia and heart failure. Using the JMH as the gold standard, the sensitivity, specificity, positive predictive value, negative predictive value and overall accuracy of CMR were 89%, 90%, 67%, 97% and 90% respectively. The Kappa agreement between the JMH criteria and CMR was 0.70. Interestingly, 4 patients had evidence of CS on CMR but did not meet the JMH criteria, while 1 patient who met JMH had no evidence of LGE.

## Conclusion

In patients with sarcoidosis, CMR is a useful diagnostic tool to determine cardiac involvement, and is able to recognize an additional 11% of patients with probable cardiac sarcoidosis. JMH criteria alone may miss one third of CS cases. While this may be worse in asymptomatic patient, including CMR in the workup of CS may improve the diagnostic yield.

